# A UK healthcare professional survey on the islet autoantibody status of children and young people with pre‐stage 3 type 1 diabetes, on behalf of the British Society for Paediatric Endocrinology and Diabetes

**DOI:** 10.1111/dme.70069

**Published:** 2025-05-23

**Authors:** Rabbi Swaby, Tabitha Randell, Jane Bowen‐Morris, Colin Dayan, Daniela Elleri, Clare Hambling, Rebecca Martin, Sarinda Millar, Pooja Sachdev, Ambika Shetty, Julia Townson, Rachel E. J. Besser

**Affiliations:** ^1^ Nuffield Department of Medicine, Centre for Human Genetics, NIHR Oxford Biomedical Research Centre University of Oxford Oxford UK; ^2^ Nottingham Children's Hospital Nottingham University Hospitals NHS Trust Nottingham UK; ^3^ Division of Infection and Immunity School of Medicine, Cardiff University Cardiff UK; ^4^ Clinical Diabetes and Metabolism School of Medicine, Cardiff University Cardiff UK; ^5^ Endocrinology and Diabetes Department Royal Hospital for Children and Young People Edinburgh UK; ^6^ Scottish Study Group for the Care of Diabetes in the Young Edinburgh UK; ^7^ Litcham Health Centre Norfolk UK; ^8^ Child and Adolescent Services University College London Hospitals NHS Foundation Trust London UK; ^9^ Daisy Hill Hospital Southern Health and Social Care Trust Newry UK; ^10^ School of Medicine University of Nottingham Nottingham UK; ^11^ Department of Paediatric Diabetes and Endocrinology The Noah's Ark Children's Hospital for Wales, Cardiff and Vale University Health Board Cardiff UK; ^12^ Centre for Trials Research Cardiff University Cardiff UK; ^13^ Department of Paediatrics John Radcliffe Hospital Oxford UK

**Keywords:** early‐stage, identification, management, monitoring, screening, type 1 diabetes

Screening research programmes around the world are identifying children and young people (CYP) with early‐stage type 1 diabetes (T1D), defined by the presence of ≥2 islet autoantibodies (IAb), before the onset of clinical disease (pre‐stage 3 T1D).^1^ It has been recognised that monitoring IAb‐positive individuals is key to observing the clinical benefits, which include a reduction in diabetic ketoacidosis and the need for hospitalisation at clinical onset, and identifying CYP eligible for immune intervention.^2^, ^3^, ^4^, ^5^ Recent international consensus guidance has provided recommendations for the monitoring of affected individuals in clinical care.^6^

Several screening research programmes exist in the United Kingdom, offering testing to first‐degree relatives and the general population.^7^ Anecdotal reports suggest that CYP may also be identified through clinical care (personal communication *R Besser*). We therefore sought to identify the numbers of children with pre‐stage 3 T1D being managed by paediatricians and how they were identified. In addition, since dropout from screening and follow‐up can be as high as 50%,^2^, ^8^, ^9^ we sought to gather information on the number of CYP with ≥1 IAb who are not on insulin, and their management, from research screening platforms as well as those who had been identified in clinical care.

We distributed an electronic survey via all 188 UK paediatric diabetes units (PDUs) between March 2024 and July 2024. Data were collected on the type of centre (district general hospital or tertiary hospital), reason for IAb testing, IAb status (single or multiple), management strategies and attitudes to sibling testing.

The survey was completed by 124/188 (66%) of PDUs contacted: 111/172 (65%) from England and Wales, 9/11 (82%) from Scotland and 4/5 (80%) from Northern Ireland. Of those PDUs who responded to the survey, 106/124 (85%) were district general hospitals and 18/124 (15%) were tertiary centres. This is similar to PDUs that did not respond (55/64 (86%) district general hospitals, and 9/64 (14%) tertiary centres). Twenty‐eight per cent of units (35/124) reported managing 145 CYP with ≥1 IAb: 41/145 (28.3%) with a single IAb, 102/145 (70.3%) with ≥2 IAb and 2/145 (1.4%) with unknown IAb status. Of the PDUs who reported managing IAb‐positive individuals, 24/35 (69%) were district general hospitals, with a median of 1 IAb‐positive child per PDU (IQR, 1–2) and 11/35 (31%) were tertiary centres, with a median of 5 IAb‐positive individuals per PDU (IQR, 2–12). Of these 35 PDUs with IAb‐positive individuals, 49% reported that CYP were identified from a clinical care setting (44% from secondary care and 5% from primary care), and 51% from a research screening programme.

The reasons units reported for IAb testing included screening as part of a research programme (39%), clinical symptoms suggestive of new‐onset diabetes (32%), family screening in secondary care (13%), non‐specific symptoms resulting in an autoimmune screen (13%) and a high glycated haemoglobin (HbA1c) from a primary care practitioner/general practitioner (GP) (3%) (Figure 1a).

**FIGURE 1:**
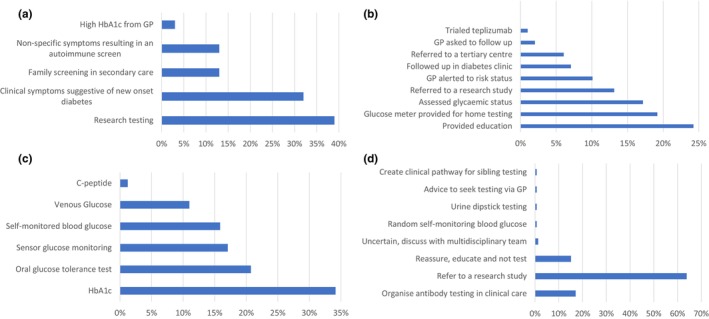
(a) The reasons reported for islet autoantibody testing in children and young people. (b) Management strategies used for children identified as islet autoantibody‐positive. (c) Methods used to assess glycaemic status. (d) Clinicians' attitudes to islet autoantibody testing in unaffected siblings of islet autoantibody‐positive children and young people. GP, general practitioner; HbA1c, glycated haemoglobin.

The strategies used to manage IAb‐positive children were broad. The most commonly used were to provide education (24%), safety netting by providing a glucose meter for home testing (19%), glycaemic assessment (17%) and referral to a research study (13%) (Figure 1b). To assess glycaemic status, a variety of tests were used, most commonly HbA1c (34%), oral glucose tolerance test (21%), sensor glucose monitoring (17%) and self‐monitored blood glucose (16%) (Figure 1c). When asked about sibling testing, most clinicians opted to refer unaffected siblings to a research study (64%). However, 18% of clinicians did support IAb testing of unaffected siblings in clinical care, with the majority (17%) stating they would organise IAb testing in a hospital setting, if requested by parents. The remainder chose not to test or refer, but instead, reassure (15%) or offer follow‐up monitoring (3%), with the remainder uncertain, seeking advice from the multidisciplinary team (1%) (Figure 1d).

This survey shows that CYP are being identified from both clinical care as well as research screening programmes across the United Kingdom. There is heterogeneity in the approach to monitoring IAb‐positive individuals. Although the larger proportion of responding PDUs were district general hospitals, tertiary centres report managing more IAb‐positive individuals per PDU. We have not surveyed GPs on their attitudes and frequency of screening and monitoring, although this survey would suggest it is low. However, in the United Kingdom, childhood diabetes is managed as a specialist service in the hospital setting, and so the monitoring of IAb‐positive children is likely to be led by specialists with experience in T1D. Some clinicians considered offering IAb testing to unaffected siblings of IAb‐positive CYP in routine clinical care, as suggested by recent American Diabetes Association (ADA) recommendations.^10^

An international consensus has recently been developed to guide monitoring. However, our finding of heterogeneity in the management strategies used for monitoring implies that a UK‐specific guideline to support both a screening and follow‐up management pathway in clinical care is urgently needed. Further, the service and cost impact of integrating screening into clinical care systems needs to be fully addressed.

## FUNDING INFORMATION

None.

## CONFLICT OF INTEREST STATEMENT

RS has received speaker honoraria from Sanofi. REJB reports acting as an independent advisor for Provent Bio and received a speaking honorarium from Sanofi, which was donated to an education research fund. TR has received speaking honoraria from Sanofi, Sandoz and Novo Nordisk. PS has received speaking honoraria from Sandoz and Novo Nordisk. CMD has lectured for or has been involved as an advisor to the following companies: Novo Nordisk, Sanofi‐Genzyme, Janssen, Servier, Lilly, AstraZeneca, Provention Bio, UCB, MSD, Vielo Bio, Avotres, Worg and Novartis. CMD holds a patent jointly with Midatech.

## Data Availability

The original survey and data presented are available on request from authors.
